# A retrospective review of leprosy diagnosed histopathologically from skin biopsy specimens in central South Africa

**DOI:** 10.4102/sajid.v41i1.802

**Published:** 2026-06-30

**Authors:** Ruben B. van Wyk, Danita L. le Grange, Liska Budding

**Affiliations:** 1 Department of Anatomical Pathology, Faculty of Health Sciences, University of the Free State, Bloemfontein, South Africa; 2 Department of Anatomical Pathology, National Health Laboratory Service, Bloemfontein, South Africa

**Keywords:** leprosy, South Africa, histopathology, diagnosis, subtypes, Ridley-Jopling classification, skin biopsy

## Abstract

**Background:**

Leprosy, caused by *Mycobacterium leprae*, remains a condition designated by the World Health Organization (WHO) as a neglected tropical disease, despite formal eradication in South Africa. Histopathology is pivotal in the diagnosis of leprosy, particularly in resource-limited settings.

**Objectives:**

The study aimed to describe the histopathological spectrum and clinical features of leprosy diagnosed on skin biopsies over a 10-year period in central South Africa.

**Method:**

A retrospective review was performed of histologically confirmed leprosy cases diagnosed from January 2015 to December 2024. Data were extracted from laboratory records and analysed descriptively.

**Results:**

Twelve patients were included (mean age 43.9 years, range 25 years – 64 years; male-to-female ratio 1.4:1). Most resided in the Mangaung Metropolitan area, with additional cases dispersed across the Free State and Northern Cape provinces. Punch biopsies predominated, most commonly obtained from the head and/or neck and the upper limbs. World Health Organization classification identified 62% of cases as multibacillary (MB). Ridley-Jopling (RJ) subtypes were mostly lepromatous leprosy (38%), while 23% of cases were indeterminate. Repeat biopsies revealed treatment response or persistent disease. Comorbidities included HIV and tuberculosis.

**Conclusion:**

Leprosy persists as a sporadic but geographically widespread disease in central South Africa, often presenting in advanced MB forms. Histopathology remains essential for diagnosis and subtyping. Strengthening clinician awareness, biopsy protocols, and treatment continuity is critical to achieving the goal of zero leprosy of the WHO.

**Contribution:**

The findings confirm that, although rare, leprosy still prevails in central South Africa. Therefore, clinicians and anatomical pathologists should consider a diagnosis of leprosy in patients with atypical or non-resolving signs and symptoms.

## Introduction

Leprosy is a chronic granulomatous disease caused by *Mycobacterium leprae* and less commonly by *Mycobacterium lepromatosis*, both obligate intracellular acid-fast bacilli (AFB).^1,2^ These organisms primarily affect the skin and peripheral nerves but may also involve the mucosa, eyes, testes and other tissues.^2^ *Mycobacterium leprae* was first identified by Gerhard Hansen in 1873, making leprosy the first human disease attributed to a specific pathogen.^3^ *Mycobacterium leprae* grows slowly, with an incubation period ranging from several months to decades. The bacterium has not yet been cultured in vitro, complicating research and diagnosis.^2,4^ Transmission is believed to occur mainly via respiratory droplets, although skin contact and zoonotic sources, particularly armadillos in some regions, have also been implicated.^2,5^ Globally, leprosy was declared ‘eliminated as a public health problem’ by the World Health Organization (WHO) in 2000, elimination being defined as a prevalence of less than one case per 10 000 population.^4^ However, this does not mean zero incidence. Leprosy has been a notifiable disease in South Africa since 1921, and records show that it was eliminated in South Africa, with a prevalence of 0.013 cases per 10 000 population in 2005.^6^ Notwithstanding, sporadic cases continue to be reported, especially in provinces such as Gauteng^7^ and KwaZulu-Natal,^8^ as evidenced by ongoing, histologically confirmed diagnoses at tertiary hospitals.^7,8^ Leprosy is one of the WHO-designated neglected tropical diseases (NTDs) and is targeted under the Global Leprosy Strategy 2021–2030.^4^ The WHO gives prominence to 22 ‘global priority countries’, which include India, Brazil, Bangladesh, Indonesia and several African nations, such as the Democratic Republic of the Congo (DRC), Ethiopia and Mozambique.^9^ India remains the country with the highest number of reported new cases, contributing over 60% of global figures, followed by Brazil and Indonesia. In Africa, the DRC and Mozambique report a relatively high burden of disease.^5^ Risk factors include close household contact with patients with multibacillary (MB) leprosy, poor socioeconomic conditions, immune suppression and residence in endemic areas.^2,5,10^ Leprosy can present as a spectrum of disease, and classification is crucial for both diagnosis and treatment. The Ridley-Jopling (RJ) classification is based on clinical, histological and immunological features, and includes the following subtypes: tuberculoid (TT), borderline tuberculoid (BT), mid-borderline (BB), borderline lepromatous (BL) and lepromatous leprosy (LL).^11,12^ Tuberculoid is characterised by robust cell-mediated immunity and localised lesions, while LL is marked by poor immunity, widespread infiltrative lesions and high bacillary load. The WHO classification simplifies the spectrum of disease for practical use and distinguishes between paucibacillary (PB) leprosy when a person presents with ≤ 5 skin lesions and negative skin smears, and MB leprosy in individuals with > 5 lesions, or any smear-positive case.^10^ Clinically, most cases of leprosy present with hypopigmented or erythematous patches, nodules, plaques, sensory loss and nerve thickening.^1,13^ Tuberculoid and BT subtypes tend to show localised lesions with loss of sensation, while LL and BL subtypes show symmetrical nodular or diffuse infiltration with systemic involvement, including polyneuropathy, ocular disease (iritis or keratitis), orchitis and upper respiratory tract symptoms. Thickened peripheral nerves and limb deformities may develop, especially in longstanding or untreated cases.^3,5,13^ The WHO diagnostic criteria specify that leprosy may be diagnosed clinically with any one of the following three findings: (1) a hypopigmented or reddish skin patch with definite loss of sensation; (2) thickened and/or enlarged peripheral nerve with associated sensory or motor deficit; or (3) the presence of AFB in slit-skin smears or skin biopsy.^10^ Histopathological examination is essential for confirming the diagnosis, especially in ambiguous or early-stage cases. Routine haematoxylin and eosin (H&E) staining can reveal key features of leprosy, but the features differ greatly depending on subtype. Tuberculoid leprosy reveals non-caseating epithelioid granulomas encapsulated by lymphocytes with occasional Langhans-type giant cells. Perineural and perivascular lymphocytic infiltrates are common. Acid-fast bacilli are rare and difficult to identify, even with special stains. Lepromatous leprosy shows sheets of foamy macrophages within the dermis. Numerous AFBs are present, especially in foamy macrophages. A plasmacytic infiltrate is commonly seen. Fite-Faraco histochemical staining is particularly useful in identifying the weakly acid-fast mycobacterial bacilli, which may be unnoticed with conventional Ziehl-Neelsen stains.^1,14^ In our laboratory, most cases of leprosy are diagnosed based on histopathological findings by evaluating the H&E stain for characteristic features suggestive of the disease. When such features are present, a Fite-Faraco stain is performed to confirm the presence of AFB. Unfortunately, no microbiological or molecular diagnostic tests, for example, polymerase chain reaction (PCR), are available in our facility. Molecular diagnostic testing with PCR is available in other centres in South Africa, while more specialised tests, including serological assays with enzyme-linked immunosorbent assay (ELISA) and lateral flow assay testing, are available internationally. Recent South African studies show a low but persistent burden of disease, often diagnosed in the late and MB stages. A retrospective review^7^ at Chris Hani Baragwanath Academic Hospital in Johannesburg, Gauteng, identified 80 confirmed cases of leprosy over a 17-year period. Multibacillary leprosy was the most common subtype, followed by LL and BL. A male predominance was identified.^7^ Similarly, a study in KwaZulu-Natal that included 194 cases over a 20-year period identified MB subtype leprosy in 90% of patients, emphasising diagnostic delays because of late presentation, inadequate access to healthcare facilities and lack of awareness or clinical suspicion.^8^ A study performed in our setting in Bloemfontein, Free State province, describing NTDs identified from 2015 to 2020, reported 72 confirmed NTDs, of which nine (12.5%) were diagnosed as leprosy. These NTDs also had a male predominance. Among those diagnosed with leprosy, the majority were reported to have PB leprosy.^15^ Several international studies reflected these patterns, with MB leprosy as the most common subtype and male predominance.^1,16,17^ Social stigma, poor socioeconomic circumstances and lack of awareness, both by health care workers and by members of the community, were identified in most cases. Together, these studies confirm the diagnostic value of histopathology and the ongoing need for surveillance, clinician training and public awareness.^18,19^ Despite formal elimination in many countries, leprosy remains a notable public health issue, especially among vulnerable populations. Its wide clinical spectrum and overlapping clinical presentations with other diseases necessitate the use of histopathology for definitive diagnosis and classification. Understanding the epidemiology and diagnostic tools is essential to timely treatment and prevention of disability. Continued reporting of new cases at referral centres in South Africa underscores the importance of clinician awareness and laboratory support. As the WHO aims towards zero leprosy, integration of histological tools into primary diagnostic pathways will remain critical. Therefore, the aim of this study was to describe the histopathological spectrum and clinical features of leprosy diagnosed on skin biopsies over a 10-year period in central South Africa.

## Research methods and design

### Study design and setting

A retrospective descriptive case series was conducted of all histologically confirmed cases of leprosy diagnosed at the Department of Anatomical Pathology, University of the Free State (UFS) and National Health Laboratory Service (NHLS) in Bloemfontein, South Africa, over a 10-year period from 01 January 2015 to 31 December 2024. The study was based exclusively on existing laboratory records, with no re-examination of tissue slides or blocks and no patient interaction.

### Data collection

All patients with a histologically confirmed diagnosis of leprosy within the specified period constituted the study population, and patients under the age of 18 years were excluded. Patients younger than 18 years of age were excluded because leprosy may present with different clinical and immunological characteristics in the paediatric population. Histopathology reports were retrieved from the NHLS TrakCare database and the NHLS Central Data Warehouse. Relevant data were extracted during the period 05 October 2025–17 October 2025, using a structured data-capture form and entered into Research Electronic Data Capture (REDCap) (REDCap Consortium; Nashville, TN, United States [US]) for secure storage and analysis. The variables that were extracted from the histopathology reports included frequency of diagnosed leprosy, patients’ age and sex, residential area, subtype according to the RJ and WHO classification systems, clinical presentation, clinical differential diagnosis, anatomical site and type of biopsy, and patient comorbidities. A pilot review of the first five confirmed cases was conducted to validate the data collection tool. These cases were included in the final data analysis.

### Statistical analysis

Descriptive statistics were applied, and categorical variables were summarised as frequencies and percentages, while continuous variables were summarised using means and standard deviations, or medians and percentiles, as appropriate. All analyses were performed by the principal investigator, Ruben B. van Wyk, following consultation with the Department of Biostatistics, UFS.

### Ethical considerations

Ethical clearance to conduct this study was obtained from the University of the Free State Health Sciences Research Ethics Committee (No. UFS-HSD2025/0952/2810). As the study used existing anonymised histopathology reports as the data source and involved no patient interaction, individual consent was waived in accordance with institutional ethics guidelines.

## Results

**FIGURE 1:**
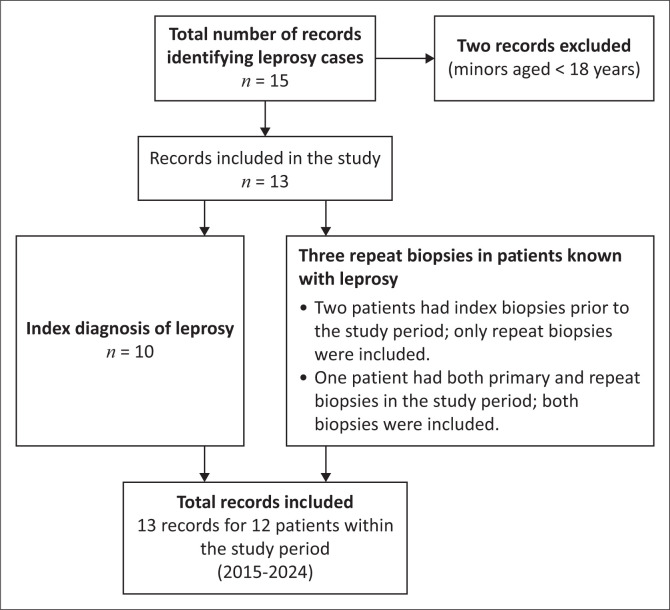
Flowchart illustrating the identification, and inclusion and exclusion process of leprosy cases.

Between January 2015 and December 2024, 15 histologically confirmed records of leprosy were identified, as represented in Figure 1. Two leprosy cases occurred in minors younger than 18 years of age and were excluded. Among the 13 included records, 10 leprosy cases were first-time diagnosis, and 3 leprosy cases represented repeat biopsies from patients known to have leprosy. As one repeat biopsy involved a patient with a first-time diagnosis during the study period, the cohort comprised 12 patients.

Table 1 presents a detailed summary of the demographic profiles, clinical features, biopsy characteristics and histopathological classification of the histologically confirmed cases of leprosy. Seven patients were male, with a male-to-female ratio of 1.4:1. The mean age of patients who had a first-time diagnosis of leprosy was 43.9 years, while the age range for the whole cohort was 25 years – 64 years. Table 2 provides an overview of the overall findings.

**TABLE 1:** Detailed summary of demographic, clinical and histopathological characteristics of leprosy cases diagnosed in central South Africa, 2015–2024.

Case number	Date of biopsy	Age (years)	Sex	First-time diagnosis and repeat biopsy	Biopsy type	Anatomical biopsy site	Classification		Clinical presentation	Clinical differential diagnosis	Comorbidities and previous medical history
							WHO	Ridley-Jopling			
1	27 August 2015	56	Female	Repeat biopsy (initial diagnosis in 2013)	Punch	Upper limb – forearm	PB	Indeterminate	Hypopigmented macule with paraesthesia.	Persistent leprosy	Previously diagnosed with multibacillary leprosy in 2013
2	26 June 2015	36	Female	First-time diagnosis	Excision	Lower limb – knee	MB	HL	Multiple nodules on elbows, knees, distal lower limbs and feet for 6 months.	Erythema elevatum diutinum, keloids, dermatofibroma, amyloidosis, nodular histiocytosis	HIV
3	23 March 2016	62	Male	Repeat biopsy (initial diagnosis in 2013)	Punch	Head, neck – eyebrow	PB	Indeterminate	Unknown	Lepromatous leprosy	Previously diagnosed with lepromatous leprosy in 2013
4	29 April 2016	48	Male	First-time diagnosis	Punch	Upper limb – forearm	MB	TT	Violaceous papules and nodules with scaling plaques. Indurated lesions on the face.	Erythema nodosum leprosum, sarcoidosis, B-cell lymphoma	HIV, TB
5	02 February 2017	57	Male	First-time diagnosis	Excision	Upper limb – hand	PB	BT	Unknown	Leprosy	-
6	08 January 2019	44	Female	First-time diagnosis	Punch	Lower limb – ankle	PB	BT	Nodules on the feet and ankles for 2 years.	Leprosy, sarcoidosis, erythema elevatum diutinum, fungal infection	-
7	26 March 2019	64	Male	First-time diagnosis	Punch	Unknown	MB	LL	Xerosis, ulcers and neuropathy, loss of cartilage tissue.	Leprosy	-
8	10 October 2019	34	Male	First-time diagnosis	Punch	Head, neck – face; Upper limb – forearm	MB	LL	Solid papules and nodules on face, neck, trunk, upper and lower limbs for 3 years.	Leprosy, sarcoidosis, cutaneous T-cell lymphoma	-
9	23 September 2022	37	Male	Repeat biopsy (initial diagnosis in 2019)	Punch	Head, neck – face	MB	LL	Multiple nodules in face with loss of sensation on extremities and generalised body pains.	Leprosy recurrence	Previously diagnosed with lepromatous leprosy in 2019
10	26 February 2024	45	Male	First-time diagnosis	Punch	Head, neck – face	MB	Indeterminate	Leonine facies	Leprosy, sarcoidosis, amyloidosis	Chronic liver disease
11	15 July 2024	27	Female	First-time diagnosis	Punch	Head, neck – nose	MB	LL	Macules, nodules and plaques on face and lower limbs for 4 years history.	Leprosy, TB, sarcoidosis, mycosis fungoides, syphilis	-
12	17 September 2024	25	Female	First-time diagnosis	Punch	Lower limb – leg	MB	LL	Previous erythematous rash and papules. Nodular lesions on upper and lower limbs. Deep bilateral lower limb ulcers.	Vasculopathy, lymphocytic vasculitis, thrombotic vasculitis	-
13	27 September 2024	59	Male	First-time diagnosis	Punch	Unknown	PB	TT	Annular plaque on face with erythematous border and central hypopigmentation for 2 years.	Granuloma faciale, cutaneous TB, leprosy, sarcoidosis	-

Note: No.8 is the patient with two biopsies performed during the study period for first time diagnosis and no.9 is the same patient repeated 3 years later.

PB, paucibacillary; MB, multibacillary; HL, histoid leprosy; TT, tuberculoid leprosy; BT, borderline tuberculoid; LL, lepromatous leprosy; TB, tuberculosis; WHO, World Health Organization.

**TABLE 2:** Overview: demographic, clinical and histopathological characteristics of leprosy cases.

Variable	Detail	*n*	%
Total cases identified	-	15	-
Cases included (≥ 18 years)	-	13	-
Adult patients included	-	12	-
Total specimens examined	-	14	-
First time diagnosis	-	10	-
Repeat biopsies	-	3	-
Male-to-female ratio	1.4:1	-	-
Mean age (index cases)	43.9 years	-	-
Age range (total cohort)	27 years – 64 years	-	-
Most frequent biopsy site	Head and neck	5	36
Most common biopsy type	Punch biopsy	11	85
WHO classification	Multibacillary	8	62
	Paucibacillary	5	39
Ridley-Jopling classification	LL	5	39
	TT	2	15
	BT	2	15
	HL	1	8
	Indeterminate	3	23
Frequent clinical differential diagnoses	Leprosy	11	-
	Sarcoidosis	6	-
	Lymphoma	3	-
Listed comorbidities	HIV	2	-
	TB	1	-
	Chronic liver disease	1	-

WHO, World Health Organization; TB, tuberculosis; LL, lepromatous leprosy; TT, tuberculoid leprosy; BT, borderline tuberculoid; HL, histoid leprosy.

**FIGURE 2:**
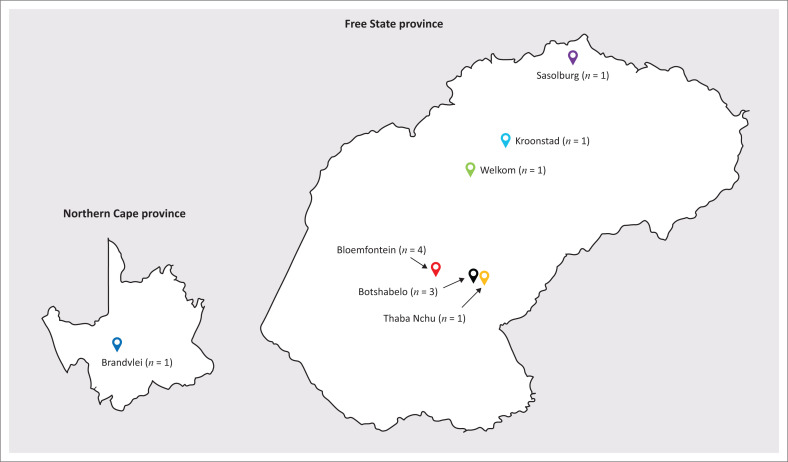
Regional distribution of leprosy cases in central South Africa, 2015–2024.

The geographical distribution of patients diagnosed with leprosy, illustrated in Figure 2, indicated that most patients resided in the greater Mangaung Metropolitan area that includes Bloemfontein (*n* = 4), Botshabelo (*n* = 3) and Thaba Nchu (*n* = 1). The remaining patients resided in other areas of the Free State province (*n* = 3) and the adjacent Northern Cape province (*n* = 1).

A total of 14 skin specimens were submitted for the 12 included patients. Two specimens were received for one patient record (Table 1, case no. 8). The most common anatomical biopsy sites were the head and neck (*n* = 5; 36%), followed by the upper limbs (*n* = 4; 29%) and lower limbs (*n* = 3; 21%), while in two patients (14%) the biopsy site was not specified. The majority were punch biopsies (*n* = 12; 86%) with excision biopsies submitted for only two cases (14%). According to the WHO criteria, cases were classified as MB (*n* = 8; 62%) and PB (*n* = 5; 39%). Based on the RJ classification, cases were designated as LL (*n* = 5; 38%), BT (*n* = 2; 15%), TT (*n* = 2; 15%) and histoid leprosy (HL) (*n* = 1; 8%), while three cases (23%) were indicated as indeterminate disease. The clinical differential diagnoses listed by referring clinicians prior to histological confirmation were diverse and often included multiple clinical diagnoses. Among the index cases, as shown in Table 1, the most frequently considered differential diagnoses were leprosy (*n* = 8) and sarcoidosis (*n* = 6), followed by lymphoma (*n* = 3), erythema elevatum diutinum (EED) (*n* = 2), amyloidosis (*n* = 2) and tuberculosis (*n* = 2). Other less frequently considered differential diagnoses included keloids, dermatofibroma, nodular histiocytosis, fungal infection, syphilis, vasculopathy, vasculitis and granuloma faciale. Comorbidities were documented in several patients, including HIV infection (*n* = 2), tuberculosis (*n* = 1) and chronic liver disease (*n* = 1), all representing states of potential immunocompromise.

## Discussion

This retrospective case series from a single public sector academic pathology service in central South Africa showed that histologically confirmed leprosy, while uncommon, persists across a broad geographic footprint and presents with a wide clinical and histopathological spectrum. The cohort had a mean age of 43.9 years at initial diagnosis. The male predominance aligned with patterns commonly described in regional and international reports and may reflect biological, behavioural or access-to-care differences.^1,7,20^

**FIGURE 3:**
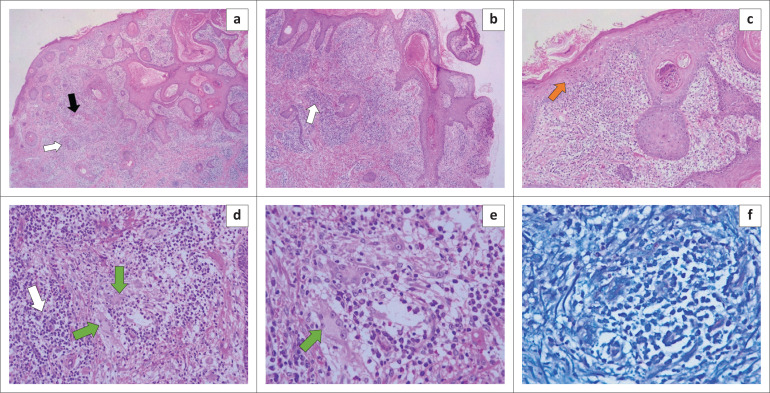
Tuberculoid leprosy: (a, b) Haematoxylin and eosin (H&E) stain showing superficial and deep dermal infiltration with non-caseating granulomas (black arrow) surrounded by numerous lymphocytes (white arrows) (a, ×2; b, ×4). (c) H&E; epidermal involvement with lymphocytic exocytosis (orange arrow) and absence of a grenz zone (×10). (d, e) H&E; perivascular granulomatous inflammation with abundant lymphocytes and Langhans-type giant cells (green arrows) (d, ×20; e, ×40). (f) Fite-Faraco stain: no acid-fast bacilli identified (×40).

**FIGURE 4:**
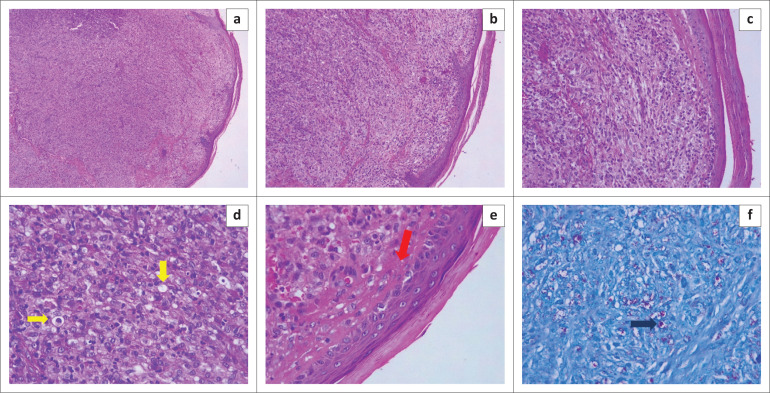
Lepromatous leprosy: (a, b, c) Haematoxylin and eosin (H&E) stain showing diffuse dermal infiltration by foamy macrophages with poorly circumscribed nodule formation and scant lymphocytic infiltrates (a, ×4; b, ×10; c, ×20). (d) H&E; sheets of foamy macrophages containing numerous intracellular acid-fast bacilli within vacuolated cytoplasm, forming Virchow cells and globi (yellow arrows) (×40). (e) H&E; narrow grenz zone beneath an intact epidermis (red arrow) overlying the foamy macrophage infiltrate (×40). (f) Fite-Faraco stain demonstrating numerous acid-fast bacilli, with conspicuous clumping within Virchow cells (blue arrow) (×40).

Most patients resided in the urban regions of the Mangaung Metropolitan Municipality, with additional single cases dispersed across several towns, suggesting low incidence but geographically diffuse transmission within the region. The dispersion of single cases across several districts underscores that leprosy in the Free State is not confined to a single hotspot; rather, sporadic cases occur province-wide, with referral into the tertiary system from multiple facilities. This distribution highlights the need for broad clinician awareness beyond specialist dermatology and neurology clinics, including at district and regional hospitals. The predominance of MB leprosy (62%) and the high proportion of LL cases (38%), based respectively on the WHO and RJ classification criteria, indicated that for many patients, a correct diagnosis was reached only when the disease was at an advanced stage. The skin specimens submitted (*n* = 14) from the included patients (*n* = 12) were predominantly punch biopsies. This type of biopsy is generally adequate when targeted to an active border, and the high proportion of MB disease in this series likely enhanced histopathologic sensitivity.^21^ Head and neck and upper limbs were the most frequent biopsy sites, with lower limbs being less common, and two specimens lacking site specification on the request forms. Although occurring at a low frequency in this study, missing site information can constrain clinicopathologic correlations. Standardised request forms for histopathological examination of skin biopsies would improve interpretability. Suggested information to be included by the requesting clinician in these standardised request forms includes the site of biopsy, lesion age, distribution of disease, sensory change and nerve involvement. The WHO categories and RJ subtypes were mostly concordant, with WHO favouring MB over PB, and RJ subtypes distributed across the spectrum from the TT pole to the lepromatous pole. The higher number of LL matched the slight shift towards MB disease, suggesting a heavier bacterial load and later diagnosis. One case was identified as HL, which is known for active bacterial growth and can occur de novo or after interrupted and/or irregular treatment.^22^ Despite the low frequency at which leprosy is diagnosed on histopathological specimens, these findings reinforce the essential role of histopathology in the diagnosis of the disease. Microscopic features complemented by Fite-Faraco staining are able to successfully confirm diagnosis, determine subtype disease, and inform treatment category. Figure 3 and Figure 4 show representative sections from two of the included cases. Figure 3 demonstrates a case of PB leprosy with TT histopathological features, including non-caseating granulomas, numerous lymphocytes and Langhans-type giant cells. Figure 4 represents a case of MB leprosy with LL histopathological features, including a diffuse infiltration of foamy macrophages and abundant bacilli on special stains.

Clinical disease patterns often aligned with histological classification, but not consistently, and this variability is acceptable along the leprosy spectrum.^23^ Localised hypoaesthetic macules or annular plaques corresponded with PB and TT presentations, whereas diffuse papulonodular disease with prominent facial involvement, neuropathy, xerosis and occasional ulceration is often reflected in MB and LL (including histoid) disease. From this perspective, several cases showed partial or atypical overlap (e.g. indeterminate histology with broad clinical spread, or MB by WHO classification with TT histological features). Such discordance is acceptable and reflects real-world heterogeneity influenced by lesion chronicity, sampling site, host immunity and treatment status and duration. Furthermore, it underscores the need to correlate clinical distribution and sensory change with histology rather than relying on any single descriptor.^23^ The duration of symptoms prior to skin biopsy was noted in only five cases, reflecting inconsistent clinical documentation. Where documented, these patients reported a prolonged duration of symptoms prior to biopsy, supporting the importance of early histopathological evaluation in suspected cases. The pre-biopsy differential diagnoses reflected the wide clinical mimicry of leprosy. Among incident presentations, clinicians most often considered leprosy itself and sarcoidosis, followed by lymphoma and tuberculosis. Less frequent single-case alternatives included EED, amyloidosis, cutaneous T-cell lymphoma and/or mycosis fungoides, keloids, dermatofibroma, nodular histiocytosis, deep fungal infection, syphilis, vasculopathy, vasculitis and granuloma faciale. This wide range of differential diagnoses could be expected, as sarcoidosis and TB share granulomatous patterns.^24^ Lymphoma can mimic lepromatous disease with diffuse dermal infiltrates. Erythema elevatum diutinum, amyloidosis and vasculitic processes present with nodules and/or plaques or ulceration, and common benign lesions (keloid and dermatofibroma) may resemble localised PB plaques.^25^ In practice, distinguishing features include sensory loss, nerve enlargement and/or tenderness, and neurotropism on histology, supported by Fite-Faraco stains and correlation with lesion distribution and chronicity – particularly important in immunocompromised patients, where patterns can be atypical and bacillary burdens higher.^26^ Three repeat biopsies were performed to assess for persistent disease, confirm treatment success, or to consider recurrence after treatment interruption linked to drug shortages. Beyond individual patient management, this signals a systems issue as uninterrupted multidrug therapy is crucial to successful outcomes and reducing transmission risk.^27^ This highlights the importance of reliable drug supply and clear follow-up plans. Despite national ‘elimination’ status by prevalence thresholds, sporadic cases continue to surface. The distribution of leprosy cases occurring in urban centres and smaller towns across the Free State province argues for sustained surveillance, periodic upskilling of primary care clinicians, and easy access to diagnostic facilities. Including leprosy in differential diagnosis checklists for chronic plaques and nodules with sensory change, and for unexplained peripheral neuropathy, may shorten time to diagnosis. In the repeat biopsies, histology captured clinically meaningful post-treatment shifts. Two patients who were initially classified as MB and LL showed downgrading on repeat histological examination to PB and indeterminate, compatible with treatment response and immune reconstitution towards the TT pole. In contrast, one patient remained MB and LL on repeat, with persistent macrophage-predominant infiltrates and a high bacillary burden, consistent with ongoing disease or relapse, and clinically aligning with the history provided of a treatment interruption because of treatment shortages. Comorbidities included HIV infection, tuberculosis and chronic liver disease, all associated with an immunocompromised state. Such conditions increase susceptibility to *M. leprae* disease and bias disease phenotypes towards the multibacillary-lepromatous spectrum by attenuating cell-mediated (Th1) responses.^28^ Morphologically, this tends to blunt granuloma formation, increase macrophage (Virchow cell) predominance, and elevate bacillary counts. Clinically, it broadens the presentation and the differential (e.g. sarcoidosis, lymphoma, TB),^29^ as reflected in this cohort. The presence of an HIV-associated histoid case further illustrates how immunodeficiency can drive atypical, nodular MB variants with high organism burdens. Together, these comorbidities both heighten disease risk and modulate histopathology, reinforcing the importance of documenting HIV status and response to antiretroviral therapy at leprosy diagnosis and during follow-up.^28,30^

### Limitations

A limitation of the study was its retrospective design and reliance on report narratives. A prospective study would be challenging given the rarity of leprosy in our setting and the low numbers of cases encountered annually. Some variables were absent from histopathology request forms, clinical details likely varied by referring clinician, and inter-observer differences in microscopic description were possible. Discrepancies were also noted in histopathology reports for leprosy skin biopsies, given the rarity of the disease. In our setting, with a very low prevalence of leprosy, most histopathologists encounter very few cases and may therefore be less familiar with the standardised terminology recommended in the literature. In a small subset of cases (*n* = 2), the original reports did not assign an RJ subtype; for these, we interpreted the documented histological features and assigned an RJ category post hoc for this study to ensure consistency. Similarly, when WHO operational categories (PB or MB) were not explicitly stated, we derived them from the available clinical information (e.g. lesion counts, nerve involvement) recorded on the request forms. Only public sector cases processed at the Department of Anatomical Pathology of the NHLS were included; private sector diagnoses are not captured. Finally, microbiological and molecular tests (e.g. slit-skin smear indices, PCR) were not available, limiting bacillary quantification to histological assessment.

## Conclusion

In this central South African series, leprosy was found to persist as a sporadic but geographically widespread disease, with a slight predominance of MB and LL forms at diagnosis and a broad range of clinical features overlapping with many other conditions. Histopathology remains definitive for confirmation and subtyping where clinical suspicion exists and ancillary tests are limited. Strengthening clinician awareness, improving diagnostic access, and ensuring treatment continuity should reduce diagnostic delay, improve outcomes and support ongoing surveillance towards the goal of zero leprosy.
